# Our Contemporaries

**Published:** 1918-07

**Authors:** 


### OUR CONTEMPORARIES

---

Editorial, Southern Medical Journal, Birmingham, Ala., May, 1918.

Surgeon-General Gorgas is sixty-three years young.

The Washington Post on May 7 is responsible for the statement that Surgeon-General Gorgas will reach the retiring age of sixty-four on October 3, 1918. Of course this does not mean that General Gorgas will be retired in October, because no one would consider retiring a man who every day is demonstrating his youth and efficiency in a position of such great responsibility as that of Surgeon-General of the United States Army during the greatest war in history.

Those who are in a position to know, regard General Gorgas as second in efficiency only to President Wilson; and they marvel at the work he has accomplished in the past year. Only a young man in vigorous health could have lived through what the General has done since the War began. Office hours begin in his office at 9 o'clock, but General Gorgas is there by 8 or 8:30, and he is one of the last to leave in the afternoon.

If all the soldiers lived the hygienic life that he follows, the Army sickness and death rate would be negligible, though it should not be forgotten that the morbidity and mortality rates among our troops are less than half those in the Spanish-American War, and are lower than those of any other army that was ever gotten together.

Of course no one has considered it possible for General Gorgas to be retired during this War. Millions of mothers and fathers and other relatives and friends of our soldiers thank God every day that General Gorgas is directing the army of doctors who are fighting diseases that are as dangerous and that are as insidious enemies to mankind as the

Huns. They feel comforted every day on realizing that Gorgas is safeguarding the health and lives of their boys.

---

**Dr. Bayard Holmes** of Chicago announces the first number of the journal called ''Dementia Praecox Studies.'' It will be published quarterly at $5.00 per year.

---

Dr. Ales Hrdlicka of the Smithsonian Institution at Washington, D. C., announces the first number of a new journal, called the ''American Journal of Physical Anthropology.'' It will be published quarterly, and will have the co-operation of the Committee on Anthropology of the National Research Council.

